# Impaired thrombin generation as a reproducible feature of bleeding disorder of unknown cause

**DOI:** 10.1016/j.rpth.2026.103451

**Published:** 2026-03-26

**Authors:** Tim Dreier, Dino Mehic, Justin Oosterlee, Helmut Haslacher, Cihan Ay, Ingrid Pabinger, Johanna Gebhart

**Affiliations:** 1 Division of Hematology and Hemostaseology, Department of Medicine I, Medical University of Vienna, Vienna, Austria; 2 Department of Laboratory Medicine, Medical University of Vienna, Vienna, Austria

**Keywords:** blood coagulation disorders, blood coagulation factors, thrombin, thromboplastin

## Abstract

**Background:**

Thrombin generation (TG) has previously been investigated in patients with bleeding disorder of unknown cause (BDUC) with discrepant results.

**Objective:**

To investigate whether previously reported impairments of TG in BDUC patients can be resproduced using a automated TG analysed at different tissue factor concentrations.

**Methods:**

In this case–control cohort study, TG was measured in patients with BDUC and age- and sex-matched healthy controls using the fully automated Ceveron S100 assay with 2 trigger reagents: reagent B (RB; very low tissue factor concentration) and reagent C-low (RC; low tissue factor concentration).

**Results:**

In total, 62 patients and 22 controls were analyzed. In both reagents, patients compared with healthy controls had longer time to peak (RB: 16.2 vs 12.8 seconds; RC-low: 10.3 vs 8.5 seconds; *P* < .001), lower peak thrombin (RB: 209.0 vs 300.7 nM; *P* < .001; RC-low: 243.6 vs 299.2 nM; *P* = .011), and lastly a lower velocity index (RB: 25.6 vs 45.9 nM/min; *P* < .001; RC-low: 34.6 vs 54.3 nM/min; *P* = .001). The lag time was only significantly prolonged in patients in RB (7.6 vs 6.1 seconds; *P* < .001), but not with RC-low (3.2 vs 3.0 seconds; *P* = .403), as was the area under the curve (RB: 2776.9 vs 3276.3 nM × min; *P* < .001; RC-low: 2824.7 vs 2842.7 nM × min; *P* = .072). Crossvalidated receiver operating characteristic analysis showed an area under the curve of 0.74 (95% CI, 0.62-0.86) for RB and 0.76 (95% CI, 0.65-0.87) for RC-low. However, there was no association between the bleeding score and any TG parameters after adjustment for factor [F]VIII and FIX activity.

**Conclusion:**

This study highlights the reproducibility of impaired TG in patients with BDUC. Given that BDUC pathophysiology remains largely unknown, these findings suggest impaired TG may represent a common underlying feature of BDUC and underscore the need for further validation in independent cohorts and clinical settings.

## Essentials

• Results on TG in BDUC are discrepant.
• We assessed TG in patients with BDUC using an automated analyzer.
• Previous results of impaired TG in BDUC were reproducible.
• Patients showed significantly lower TG potential than healthy controls.

## 1 Introduction

The diagnosis of a bleeding disorder of unknown cause (BDUC) reflects normal results on in-depth hemostatic investigations, excluding known bleeding disorders, such as von Willebrand disease, platelet function defects, and coagulation factor deficiencies, despite a clinically relevant bleeding tendency. Importantly, patients with BDUC exhibit clinical characteristics and bleeding phenotypes similar to those of individuals with established bleeding disorders, and bleeding assessment tools (BATs) have failed to identify bleeding symptoms or phenotypes specific to patients with BDUC [1,2].

The pathomechanisms underlying the bleeding tendency in BDUC have remained elusive despite increased scientific interest and growing research efforts. To address this gap, global assays of hemostasis have been explored as research tools to detect subtle or heterogeneous disturbances in coagulation among patients with BDUC, which may elude conventional testing [3]. Thrombin generation (TG) assays quantify a patient’s *in vitro* capacity to generate thrombin using fluorogenic methods after activation with tissue factor (TF) and phospholipids [4].

Previously, we have reported significantly reduced TG in 382 patients from the Vienna Bleeding Biobank (VIBB) compared with 100 age- and sex-matched healthy controls using a manual TG assay [5]. Other investigations have produced inconsistent results, including studies employing fully automated TG platforms in different cohorts [[5], [6], [7], [8]]. However, whether automated TG approaches can yield findings comparable with manual assays in our BDUC cohort remains unclear. Therefore, building on the inconclusive literature, we aimed not only to replicate Hofer et al.’s [5] findings in an independent BDUC cohort but also to determine whether similar impairments in TG can be demonstrated using an automated analyzer. Furthermore, we directly compared 2 TG reagents with different TF concentrations to assess reagent-dependent variability. We hypothesize that impaired TG is a reproducible feature of BDUC and can be detected using automated TG methodology, which minimizes preanalytical variability and benefits from improved assay standardization.

## 2 Methods

BDUC was defined as the lack of an established bleeding disorder (eg von Willebrand disease, platelet function defects, or coagulation factor deficiencies) in a patient with a clinically relevant bleeding tendency, as per the recent International Society on Thrombosis and Haemostasis (ISTH) Scientific and Standardization Committee communication [3]. Patients with BDUC have been recruited into the VIBB, an ongoing, single-center, prospective cohort study conducted at the hemostasis outpatient department of the Medical University of Vienna, since its establishment in 2009. Importantly, stringent inclusion and exclusion criteria are applied to patients, as published previously [9]: patients must report a clinically relevant bleeding tendency without a prior diagnosis and must not have undergone recent surgery or childbirth, be pregnant, or have an acute-phase reaction. Exclusion criteria include severe renal or hepatic disease, active malignancy, use of anticoagulant or antiplatelet agents (including nonsteroidal antiinflammatory drugs), and thrombocytopenia (< 100 × 10^9^ platelets/L).

Patients with BDUC from the VIBB (EC Nr. 206-2009) were analyzed and compared with age- and sex-matched healthy controls (EC Nr. 039-2006) [5,10]. Both studies are approved by the ethics committee of the Medical University of Vienna and are conducted in accordance with the Declaration of Helsinki and its amendments. All patients with BDUC without prior TG investigations included in the VIBB between January 2022 and August 2023 were eligible, and no further specific selection criteria were applied to this cohort. During this timeframe, 133 patients were included into the VIBB, of whom 63 patients (47%) were classified as having BDUC according to recently published guidelines [3]. One patient with BDUC was excluded due to technical issues in TG measurement. Bleeding severity in patients with BDUC and healthy controls was quantified using the ISTH-BAT and the cutoffs of the ISTH-BAT, specific for sex in male patients and sex and age in female patients [3,11]. All potential healthy controls were screened for occult bleeding symptoms using the Vicenza bleeding score and not recruited in case of a suspected bleeding disorder (either due to an elevated score or their individual bleeding phenotype). All other inclusion and exclusion criteria for healthy controls are comparable with those of the VIBB.

In this study, we measured TG using the fully automated Ceveron S100 analyzer (Technoclone) using the Reagent B (RB; Technoclone) and Reagent C-low (RC-low; Technoclone) as triggers. Analyses were performed in platelet-poor plasma from patients and controls. Upon blood drawing after study inclusion, all samples are sent to the MedUni Wien Biobank within 1 hour for immediate processing (for platelet-poor–citrated plasma: centrifugation at > 1600*g* for 15 minutes at room temperature) and long-term storage at −80 °C in the MedUni Wien Biobank according to standard operating procedures in an ISO 9001–certified environment [12]. All samples used in this experiment were retrieved fresh from long-term storage and thawed only immediately before measurement. According to the manufacturer, both reagents have comparably low and similar concentrations of phospholipids, while the concentration of TF is higher in RC-low than in RB [13]. According to manufacturer data specific to the Ceveron, RB is recommended for TG measurement in patients with bleeding tendencies, while RC-low is advised in patients with thrombophilic tendencies [14]. TG is evaluated using 5 parameters: lag time (time from start of test to thrombin burst in minutes), time to peak (time to maximal thrombin concentration in minutes), peak thrombin (maximum thrombin concentration in nanomolars), velocity index (a composite parameter of TG kinetics in nanomolars per minute), and area under the curve (AUC, representative of the endogenous thrombin generated during the entire test in nanomolars × minutes).

Continuous and ordinal variables are reported as median and IQR, and nominal variables as counts and percentages. Pairwise comparisons between patients with BDUC and controls were performed using the Wilcoxon rank-sum test, the *t*-test or the chi-squared test, with Fisher exact test applied when expected cell counts were ≤5. Pearson correlation coefficient was applied to evaluate the relationship between TG parameters obtained with different reagents. The strength of the correlation was defined as follows: weak (*r* = 0.2-0.4), moderate (*r* = 0.4-0.6), strong (*r* = 0.6-0.8), and very strong (*r* = 0.8-1.0). The Bonferroni–Holm correction was used to correct for multiple testing. Linear regression models, adjusted for factor [F]VIII and FIX activity, were calculated to assess the associations between the ISTH-BAT score and TG parameters. For the ISTH-BAT score cutoff, a logistic regression model was used. Logistic regression was used to evaluate the discriminative ability of TG parameters between patients with BDUC and controls. Principal component analysis addressed multicollinearity among the 5 parameters, with elastic net regularization providing additional protection against overfitting given the limited sample size (events-per-variable ratio = 4.4). Model selection via 5-fold crossvalidation repeated 10 times evaluated 1 to 3 principal components, penalty values (*λ*: 10^−3^ to 10), and mixing parameters (α: 0 = ridge; α: 0.25, 0.5, or 0.75 = elastic net; α: 1 = Lasso). All preprocessing occurred within crossvalidation folds to prevent data leakage. Performance was assessed using area under the receiver operating characteristic (ROC) curve with bootstrap (*n* = 2000) CIs. Final predictions were averaged across repeats to obtain robust probability estimates. Operating characteristics were determined at Youden J threshold. Analyses were conducted using R software (version 4.5.2; R Foundation for Statistical Computing) [15] with tidymodels [16] and pROC [17] packages.

## 3 Results and Discussion

In total, 62 patients with BDUC and 22 healthy controls were analyzed. There were no significant differences between patients with BDUC and controls in terms of age, proportion of female individuals or prevalence of blood group O (Table 1). While the activated partial thromboplastin time, prothrombin time, fibrinogen, and FVIII activity were similar between patients and controls, the FIX activity was significantly lower in patients with BDUC (98% in patients and 108% in controls; *P* = .002), although within the normal range. The median ISTH-BAT score in patients with BDUC was 5 (IQR, 3-8) and 0 (IQR, 0) in healthy controls. Thirty-five patients (56%) had an ISTH-BAT score above the age- and sex-specific cutoff.

**Table 1:** Patient and control characteristics.

Parameter	Measurement method	Patients with BDUC	Healthy controls	Significance (BHC)
Cohort (*n*)	NA	62	22	—
White ethnicity	NA	62 (100)	22 (100)	—
Female sex	NA	56 (90%)	19 (86%)	.691^Fisher^ (NS)
Blood group O	NA	26 (43%)	7 (39%)	.992^chi-squared^ (NS)
Age (y)	NA	40 (29-52)	40 (29-45)	.752^Wilcoxon^ (NS)
Bleeding score^a^ (AU)	ISTH-BAT	5 (3-8)	0	NA
Above score cutoff	ISTH-BAT	35 (56)^a^	0	NA
APTT (s)	APTT-STA (coagulometric)	35.9 (33.4-37.2)	34.3 (32.7-35.1)	.098^Wilcoxon^ (NS)
Prothrombin time (%)	coagulometric	92 (85-98)	96 (89-99)	.256^*t*-test^ (NS)
Fibrinogen (mg/dL)	Clauss (coagulometric)	292 (259-333)	306 (259-343)	.692^Wilcox^ (NS)
Factor VIII activity (%)	Photooptical-coagulometric	126 (101-151)	130 (104-148)	.562^*t*-test^ (NS)
Factor IX activity^b^ (%)	Photooptical-coagulometric	89 (78-102)	108 (99-128)	**.002**^*t*-test^ (**<.05**)
Factor XI activity^c^ (%)	Photooptical-coagulometric	104 (86-118)	108 (99-128)	.309^*t*-test^ (NS)
Factor XIII activity^d^ (%)	Photometric	144 (108-164)	127 (103-139)	.077^*t*-test^ (NS)
Von Willebrand factor antigen (%)	Latex-agglutination	100 (88-117)	99 (94-112)	.996^Wilcoxon^ (NS)
Von Willebrand factor-ristocetin activity (%)	Platelet-agglutination	99 (84-130)	94 (80-119)	.741^Wilcoxon^ (NS)
Antiplasmin activity (%)	Chromogenic	99 (93-104)	98 (94-104)	.600^*t*-test^ (NS)

Values are given as *n* (%) or median (IQR). Values highlighted in bold indicate statistical significance.

APTT, activated partial thromboplastin time; AU, arbitrary unit; BDUC, bleeding disorder of unknown cause; BHC, Bonferroni–Holm correction; chi-squared, chi-squared test; Fisher, Fisher exact test; NS, nonsignificance; Wilcoxon, Wilcox rank-sum test.

a Using the ISTH-BAT.

b Factor IX activity not documented in 1 patient and 1 control.

c Factor XI activity not documented in 3 patients and 1 control.

d Factor XIII activity not documented in 4 patients and 1 control.

In patients with BDUC, TG RB and RC-low correlated weakly for velocity index (*r* = 0.31; *P* = .013) and lag time (*r* = 0.38; *P* = .002); moderately for peak thrombin (*r* = 0.45; *P* < .001) and time to peak (*r* = 0.51; *P* < .001); and strongly for the AUC (*r* = 0.61; *P* < .001).

The results of the TG assays are reported in Table 2. Overall, comparison of TG between patients with BDUC and controls showed significantly impaired TG in patients with BDUC. These alterations were similar between the different reagents, however, more pronounced in TG RB. In detail, patients with BDUC had a longer lag time than healthy controls, with a significant difference in TG RB (7.7 and 6.1 minutes; *P* < .001), while there was no difference in TG RC-low (3.2 and 3.0 minutes; *P* = .403). The time to peak was significantly longer in patients with BDUC than healthy controls in both TG RB (16.3 and 12.8 minutes; *P* < .001) and TG RC-low (10.3 and 8.5 minutes; *P* < .001). Peak thrombin was lower in patients than that in controls in TG RB (208.0 and 300.7 nM; *P* < .001) but similar using RC-low after the correction for multiple testing (243.6 and 299.2 nM; *P* = .011). The velocity index was significantly lower in patients with BDUC than controls measured with both RB (25.2 and 45.9 nM/min; *P* < .001) and RC-low (34.6 and 54.3 nM/min; *P* = .001), and the AUC was significantly lower in patients with BDUC than that in healthy controls measured by RB (2765.7 and 3276.3 nM × min; *P* < .001), while it was lower but not significantly different when measured by RC-low (2824.7 and 2842.7 nM × min; *P* = .072).

**Table 2:** Thrombin generation results in patients with BDUC and healthy controls.

Parameter	BDUC patients	Healthy controls	Significance (BHC)
Cohort (*n*)	62	22	**—**
Reagent B			
Lag time (min)	7.7 (6.7-9.3)	6.1 (5.7-6.9)	**<.001**^*t*-test^ (**<.05**)
Time to peak (min)	16.3 (14.0-19.1)	12.8 (11.3-13.7)	**<.001**^*t*-test^ (**<.05**)
Peak thrombin (nM)	208.0 (157.6-307.8)	300.7 (255.2-397.3)	**<.001**^Wilcoxon^ (**<.05**)
Velocity index (nM/min)	25.2 (15.8-40.7)	45.9 (37.8-66.4)	**<.001**^Wilcoxon^ (**<.05**)
AUC (nM × min)	2765.7 (2234.0-3175.7)	3276.3 (2784.7-3632.7)	**<.001**^*t*-test^ (**<.05**)
Reagent C-low			
Lag time (min)	3.2 (2.9-3.7)	3.0 (2.6-3.4)	.403^*t*-test^ (NS)
Time to peak (min)	10.3 (9.0-11.6)	8.5 (7.6-9.7)	**<.001**^*t*-test^ (**<.05**)
Peak thrombin (nM)	243.6 (192.7-296.5)	299.2 (233.3-351.4)	**.011**^*t*-test^ (NS)
Velocity index (nM/min)	34.6 (24.5-50.2)	54.3 (38.0-71.0)	**.001**^Wilcoxon^ (**<.05**)
AUC (nM × min)	2824.7 (2492.1-3065.4)	2842.65 (2708.55-3216.4)	.072^*t*-test^ (NS)

Values are median (IQR). Values highlighted in bold indicate statistical significance.

AUC, area under the curve; BDUC, bleeding disorder of unknown cause; BHC, Bonferroni–Holm correction; NS, nonsignificance; Wilcoxon, Wilcox rank-sum test.

As FIX activity differed significantly between patients and controls, differences in TG were adjusted accordingly. In TG RB, the statistical differences prevailed for the lag time, time to peak, peak thrombin, and AUC, but not the velocity index. In TG RC-low, time to peak and the velocity index were still significantly different after the adjustment, while the difference in peak thrombin lost significance.

Next, TG with RB or RC-low was evaluated for its ability to discriminate between patients and controls using multivariable logistic regression and ROC analysis. The optimal model configuration for RB used 2 principal components with elastic net regression (λ = 0.127, α = 0.75), achieving an AUC of 0.738 (95% CI, 0.619-0.858). RC-low performed similarly with 3 principal components and Ridge regression (λ = 0.0018; α = 0), yielding an AUC of 0.760 (95% CI, 0.647-0.872) (Figure A). No significant difference was observed between reagents (DeLong test, *P* = .862).

**Figure:**
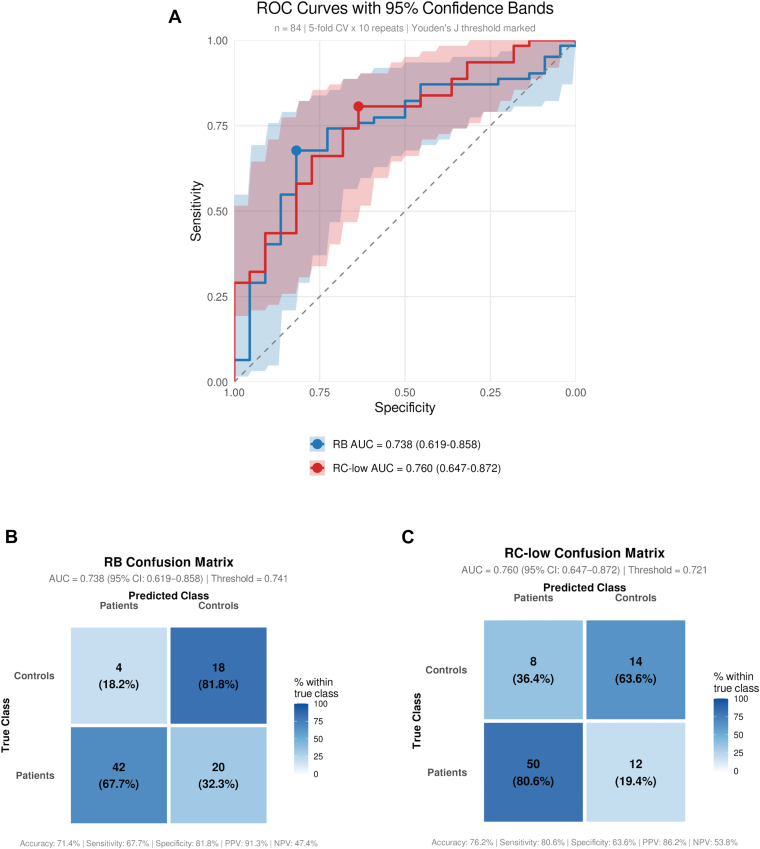
(A) ROC–AUC analysis of thrombin generation parameters after primary component analysis. Youden J (highlighted in the plot) shows the optimal threshold of the predicted probability and was used to create the confusion matrices. (B) Confusion matrix (at Youden J) of the model including TGA parameters measured with RB. (C) Confusion matrix (at Youden J) of the model including TGA parameters measured with RC-low. AUC, area under the curve; NPV, negative predictive value; PPV, positive predictive value; RB, reagent B; RC, reagent C; ROC, receiver operating characteristic.

At optimal thresholds determined by Youden J statistic (0.741 for RB; 0.721 for RC-low), RB demonstrated an accuracy of 71.4% (67.7% sensitivity and 81.8% specificity) (Figure B), while RC-low showed an accuracy of 76.2% (80.6% sensitivity and 63.6% specificity) (Figure C) based on averaged predictions across crossvalidation repeats. High crossvalidation variability (coefficient of variation = 15%) reflected the inherent challenges of modeling with limited sample size.

Our data demonstrating impaired TG in patients with BDUC confirm our previous findings from a larger cohort of 382 patients with BDUC enrolled in the VIBB, in whom TG was assessed using the Technothrombin assay (Technoclone) [5]. In that study, and in line with the current findings, patients with BDUC had a longer lag time and time to peak, lower peak thrombin, a lower velocity index, and finally, a lower AUC. However, TG has been studied in different cohorts of patients with BDUC and showed conflicting results. Differences in patient recruitment and inclusion criteria, the methodology applied to assess TG, and preanalytical or analytical variability (eg, TF concentration, phospholipid composition, or calibration methods) have been discussed as potential explanations for these discrepancies [7].

Veen et al. [6] assessed 121 patients with BDUC and 76 healthy controls using the calibrated automated thrombogram (CAT; Diagnostica Stago) and identified a prolonged lag time as the only significant difference between the groups [6]. In line, Cornette et al. [18] reported 62.7% patients with BDUC with abnormalities in TG (defined as any parameter outside the reference range) when measured using the ST Genesia TG assay (Diagnostica Stago) and a similarly high proportion of 69.5% of patients with BDUC as abnormal in TG measured using the CAT (Diagnostica Stago) [18]. In contrast, an analysis of patients with BDUC included in the predecessor study of the VIBB showed no difference in TG parameters between 101 patients with BDUC and 102 age- and sex-matched healthy controls using the Technothrombin assay (Technoclone) [8]. Likewise, Alves et al. [19], also using the Technothrombin assay, reported similar TG parameters in an independent cohort of 45 patients with BDUC and 50 healthy controls [19].

Among the various methodological factors, TF concentration in the trigger reagent appears to be of particular importance in TG measurements and may have contributed to the discrepant findings reported in BDUC [20]. Both our data, comparing 2 reagents with differing TF concentrations, and previously published studies underscore this effect. We observed significant differences between patients with BDUC and controls with both reagents; however, the discrepancies were more pronounced at lower TF concentrations (RB). Specifically, patients with BDUC exhibited prolonged lag times and reduced AUC, resulting in significant abnormalities in RB. These findings are consistent with the work of Thomas et al. [7], who likewise demonstrated longer lag times and reduced AUC at lower TF concentrations (1 vs 5 pM), together with a higher frequency of abnormalities in patients with BDUC assessed with low TF concentrations using a CAT system (Thermo Fisher Scientific) [14]. Collectively, these results emphasize key methodological challenges in TG testing, particularly the strong dependence of assay outcomes on TF concentration, which complicates the interpretation and comparability of findings across studies [5,7].

Lastly, we analyzed whether the bleeding severity in patients with BDUC was associated with alterations in TG. In multivariable regression models (Table 3), no associations were observed between the ISTH-BAT and any TG parameters after the adjustment for multiple testing. Although impaired TG capacity may represent a stable characteristic in a subset of patients with BDUC, its clinical expression is likely context dependent and influenced by additional factors beyond coagulation alone. Moreover, the reliance of the ISTH-BAT on retrospective symptom reporting and categorical thresholds, such as size and number of hematoma or duration of epistaxis, may limit its sensitivity for detecting subtle laboratory-phenotype associations. Taken together, the clinical utility of TG in patients with BDUC remains to be consecutively shown. As our study mainly investigated TG as a feature of the underlying BDUC pathomechanism, it was likely underpowered to predict a complex outcome such as the ISTH-BAT and should be interpreted with this limitation in mind. The prognostic value of TG for future bleeding risk, especially after hemostatic challenges such as surgery or childbirth, is unknown and warrants longitudinal investigations.

**Table 3:** Associations between TG parameters and the ISTH-BAT score and its cutoff.

Predictor	Outcome	
	ISTH-BAT score linear regression, β (95% CI); *P*	ISTH-BAT cutoff logistic regression, OR (95% CI); *P*
Reagent B (*n* = 62 patients with BDUC)		
Lag time (per 1-min increase)	0.20 (−0.26 to 0.66); .394	1.28 (0.97-1.75); .099
Time to peak (per 1-min increase)	0.14 (−0.12 to 0.39); .295	1.15 (0.98-1.37); .092
Peak thrombin (per 20-nM increase)	−0.05 (−0.22 to 0.13); .600	0.96 (0.86-1.06); .432
Velocity index (per 5-nM/min increase)	−0.05 (−0.22 to 0.12); .542	0.95 (0.85-1.05); .395
AUC (per-100 nM × min increase)	−0.03 (−0.19 to 0.13); .700	0.98 (0.89-1.07); .666
Reagent C-low (*n* = 62 patients with BDUC)		
Lag time (per 1-min increase)	1.30 (−0.09 to 2.70); .068	**2.59 (1.08-6.96); .043;** BHC: NS
Time to peak (per 1-min increase)	0.51 (−0.02 to 1.04); .059	**1.43 (1.02-2.09); .049;** BHC: NS
Peak thrombin (per 20-nM increase)	−0.24 (−0.50 to 0.03); .076	0.85 (0.71-1.00); .063
Velocity index (per 5-nM/min increase)	−0.24 (−0.52 to 0.03); .084	0.85 (0.70-1.01); .075
AUC (per-100 nM × min increase)	−0.16 (−0.41 to 0.10); .223	0.91 (0.77-1.05); .229

Linear and logistic regressions were adjusted for the activities of factor [F]VIII and FIX. Values highlighted in bold indicate statistical significance.

AUC, area under the curve; β, estimate of linear regression; BDUC, bleeding disorder of unknown cause; BHC, Bonferroni–Holm correction; ISTH-BAT, International Society on Thrombosis and Haemostasis—bleeding assessment tool; NS, nonsignificance; OR, odds ratio.

Our study is not without limitations. Regarding TG testing, the exact concentrations of TF and phospholipids in the trigger reagents used on the Ceveron system are not disclosed by the manufacturer, which may affect the generalizability of our results. Furthermore, our TG results were not standardized using the commercially available normal pooled plasma for the Ceveron system, which may limit direct comparability with studies that used such standardization. With respect to the diagnostic workup, routine measurement of FII, FV, FVII, and FX was not performed in all patients with BDUC, and we therefore cannot completely exclude that rare mild or monoallelic coagulation factor deficiencies may have contributed to the bleeding phenotype in individual cases.

Despite these limitations, this study confirms and extends previous findings on impaired TG in patients with BDUC by reproducing results from Hofer et al. [5] in an independent cohort using a different automated assay. A particular strength is the assessment of new, consecutively recruited patients with BDUC and controls using identical inclusion, exclusion, and diagnostic criteria, enabling reliable comparison and validation of the previous study [5]. Despite methodological differences and nonoverlapping populations, our results consistently demonstrated significant TG alterations in patients with BDUC compared with controls, supporting the hypothesis that BDUC involves measurable hemostatic disruption as part of its pathophysiology. Underlying mechanisms of impaired TG in BDUC remain unknown, but natural anticoagulants such as increase of TF pathway inhibitor and activated protein C levels in plasma might contribute to our observations [21,22].

Importantly, our study also highlights key methodological challenges that affect the interpretation of TG results across different studies. The outlined discrepancies between studies using the same assay platform underscore the complexity of TG as a biomarker and the need for rigorously standardized protocols in both sample processing and analysis. Moreover, our inability to detect an association between TG and the total ISTH-BAT score further emphasizes the limitations of current clinical tools in capturing subtle functional disorders in coagulation.

Reproducibility is essential in poorly defined conditions such as BDUC, where diagnostic uncertainty and clinical heterogeneity are inherent. Our study provides valuable evidence toward a reproducible laboratory phenotype, reinforcing that impaired TG may reflect a poorly understood pathomechanism. Future research should evaluate TG in independent BDUC cohorts across different populations and settings. Consistent identification of coagulation abnormalities may ultimately guide development of more targeted diagnostic and therapeutic strategies for patients with BDUC.
